# A Systematic Review of School-Based Behavioral Interventions and the Symbolic Labor of Inclusion for Children with Chronic Illness

**DOI:** 10.3390/healthcare13161968

**Published:** 2025-08-11

**Authors:** Efthymia Efthymiou, Dimitra V. Katsarou, Maria Sofologi, Kalliopi Megari, Soultana Papadopoulou, Evangelos Mantsos, Salma Daiban

**Affiliations:** 1 College of Interdisciplinary Studies, Zayed University, Abu Dhabi P.O. Box 144534, United Arab Emirates; 2 Department of Preschool Education Sciences and Educational Design, University of the Aegean, 85100 Rhodes, Greece; d.katsarou@aegean.gr; 3 Psychology Laboratory, Department of Early Childhood Education, School of Education, University of Ioannina, 45110 Ioannina, Greece; m.sofologi@uoi.gr; 4 Department of Psychology, City College, University of York, Europe Campus, 54622 Thessaloniki, Greece; kmegari@psy.auth.gr; 5 Department of Speech and Language Therapy, University of Ioannina, 45110 Ioannina, Greece; papasoul@uoi.gr; 6 Department of Physical Education and Sport Science, University of Thessaly, 42100 Trikala, Greece; v.mantsos@hotmail.com; 7 College of Medicine and Health Sciences, United Arab Emirates University, Al Ain P.O. Box 15551, United Arab Emirates; sdaiban@uaeu.ac.ae

**Keywords:** chronic illness, inclusive education, systematic review, behavioral intervention, symbolic inclusion, disability studies, pedagogy of care

## Abstract

**Background:** Chronic illness affects children’s health and disrupts the spatial and temporal aspects of schooling by complicating attendance, interrupting learning routines, and exposing institutional rigidity. While many educational systems treat chronicity as an exception to be managed, this review reconceptualizes it as a pedagogical and symbolic challenge to normative assumptions about inclusion, care, and participation. **Objective:** To systematically examine how school-based behavioral and psychosocial interventions support children and adolescents with chronic health conditions (CHCs) in inclusive educational settings and to analyze what these interventions reveal about institutional practices of care and recognition. **Methods:** Following PRISMA 2020 guidelines, we conducted a systematic search across five databases, PubMed, ERIC, PsycINFO, Scopus, and Web of Science, for studies published between January 2010 and April 2025. Of 420 records screened, 28 studies met inclusion criteria. Eligible studies reported on school-based interventions for students aged 5–18 with chronic conditions. Methodological quality was appraised using the Cochrane Risk of Bias 2 tool (for RCTs) and the Joanna Briggs Institute checklist (for quasi-experimental designs). Findings were synthesized narratively and thematically. **Results:** The included studies addressed asthma, attention-deficit/hyperactivity disorder (ADHD), diabetes, epilepsy, autism, cancer, and food allergies. Interventions ranged from nurse-led management and teacher training to peer education and executive function coaching. Most reported improvements in symptom control, school attendance, academic performance, and psychosocial wellbeing. Several studies also demonstrated how interventions reshaped institutional routines and distributed responsibility for care, challenging rampant assumptions about autonomy, ability, and normativity. **Conclusions:** School-based interventions for chronic illness operate as health strategies and as symbolic and structural enactments of inclusion. When designed relationally, they modulate schools into responsive institutions where care is integrated in everyday pedagogical and organizational practices. Future research prioritizes longitudinal studies, underrepresented contexts, and the active participation of youth in shaping interventions.

## 1. Introduction

Chronic health conditions are prevalent among school-aged children, affecting an estimated 15–20% of students across Western countries [1,2]. These conditions, e.g., asthma, diabetes, epilepsy, attention-deficit/hyperactivity disorder (ADHD), and other long-term illnesses, exert significant educational, social, and emotional consequences. Students managing such conditions face elevated risks of absenteeism, academic underachievement, social isolation, and mental health challenges compared to their healthy peers [3,4]. Despite these realities, schools are rarely designed to accommodate the temporal disruptions, social visibility, and emotional fluctuations inherent to chronic illness. Educational systems tend to operate on assumptions of stable attendance, linear progression, and consistent performance, norms that chronically ill students may struggle to meet [2]. This disconnect can result in exclusion, stigma, or low expectations, because students’ realities diverge from institutional norms [3].

Students with chronic illnesses frequently report poorer school experiences and lower life satisfaction than their peers [2,5]. According to a large European study, there are three distinct profiles of school experience among these students, only one of which was broadly positive [2]. In systems where chronically ill youth are integrated into mainstream schools without adequate support, negative experiences are more likely. This signals that inclusion in name alone is insufficient, as inclusive schooling needs to be accompanied by structural and relational supports.

The psychosocial conditions of the school environment, such as perceived academic pressure, teacher responsiveness, and peer support, play a decisive role in student wellbeing [4,5]. Evidence from Sweden’s Health Behavior in School-aged Children (HBSC) surveys shows that high academic demands are consistently associated with diminished mental health, while strong social support correlates with better outcomes [4]. Importantly, peer support can buffer the effects of stress and prevent the marginalization that chronically ill students often face [3].

Negative social experiences, e.g., bullying, stigma, or exclusion, are detrimental to these students. Such experiences may stem from visible symptoms, frequent absences, or misunderstanding by staff and peers [3]. A socio-ecological approach is necessary to support chronically ill children that engage the individual, and the school as a social and institutional system.

At the system level, factors such as national inclusion policies, funding, school nurse availability, and professional development opportunities for educators enable schools to respond effectively [6]. In well-resourced systems, students are more likely to receive flexible accommodations, individualized learning plans, and access to health professionals. In contrast, under-resourced settings may lack even basic supports [7]. Cross-national differences in school satisfaction among chronically ill students reflect these structural disparities [2].

Schools have become sites for health intervention, with a growing evidence base demonstrating the cost-effectiveness of school-based programs targeting chronic diseases [8]. Interventions addressing asthma management, diabetes education, and mental health promotion have shown promise, especially when they are targeted, evidence-based, and embedded within broader support systems. However, not all programs are equally effective as universal mental health interventions and might yield mixed results [8]. This stresses the need for thoughtful design and ongoing evaluation of school health programs.

In parallel, research highlights the importance of training school personnel and building intersectoral partnerships [1]. Educators may feel underprepared to support students with health needs. Equipping them with clinical knowledge, communication strategies, and emotional support skills is critical. Moreover, partnerships with healthcare providers, families, and community agencies may provide coordinated care models that benefit students and schools [1].

A holistic, multi-component model that integrates curriculum flexibility, peer relationships, physical activity, and extracurricular engagement is needed [7]. For chronically ill students, success depends on health management and on belonging, participation, and meaningful engagement in school life. A comprehensive approach rooted in the World Health Organization’s Health Promoting Schools framework might align academic and health priorities in sustainable ways.

This review is grounded in critical perspectives from special and inclusive education that frame schooling as a disciplinary institution where time, space, and bodies are regulated [9]. Drawing from Freire and Slee, we view inclusion as a political project that challenges structures of marginalization [9,10]. Noddings’ ethics of care forefronts relational practices in education [11], while Lundy and McEvoy’s participatory model emphasizes student agency as fundamental to inclusive practice [12]. These conceptual orientations are complemented by key international policy frameworks such as the Warnock Report in 1978 and the Salamanca Statement and Framework for Action on Special Needs Education by UNESCO in 1994, which have shaped global discourse on inclusive education and continue to guide national efforts toward equitable schooling for all learners, including those with chronic conditions.

To guide this review, we pose the following research question: “*How do school-based behavioral and psychosocial interventions support children and adolescents with chronic health conditions in inclusive educational settings, across health, academic, and psychosocial domains?*”.

We also address three sub-questions:

1. What behavioral or psychosocial interventions have been implemented in school settings to support students with chronic health conditions?
2. What health, academic, and psychosocial outcomes have these interventions produced?
3. How do these interventions reflect or resist prevailing educational assumptions about ability, participation, and normativity?

This review aims to reframe inclusion as a dynamic process of institutional care, negotiation, and transformation.

## 2. Materials and Methods

### 2.1. Review Design and Registration

The review is reported according to the Preferred Reporting Items for Systematic Reviews and Meta-Analyses (PRISMA 2020) guidelines [13] to ensure transparent and complete reporting. However, the PRISMA checklist is not a tool for quality appraisal. Instead, the methodological quality of included studies was evaluated using the Cochrane Risk of Bias 2 (RoB 2) tool for randomized controlled trials and the Joanna Briggs Institute (JBI) Checklist for quasi-experimental studies. The protocol was not prospectively registered in a public repository (see Section 4.4 and Section 5). All methodological steps, including inclusion criteria, search strategy, and data extraction protocols, were predefined and documented internally.

This review addressed the primary research question, “*How do school-based behavioral and psychosocial interventions support children and adolescents with chronic health conditions in inclusive educational settings, across health, academic, and psychosocial domains?*”. The PECO framework was applied to develop the search strategy and inclusion criteria, as outlined below.

### 2.2. Research Questions and PECO Framework

The guiding research question was formulated using the PECO framework (Population, Exposure, Comparator, Outcome):

- Population: School-aged children and adolescents (5–18 years) with chronic health conditions;
- Exposure: School-based behavioral or psychosocial interventions, e.g., nurse-led programs, peer education, executive functioning training;
- Comparator: Not applicable (non-meta-analytic synthesis); pre/post or control group comparisons used where available;
- Outcomes: Health-related outcomes, e.g., symptom reduction, psychosocial functioning; e.g., peer relations, stigma, academic engagement; e.g., attendance, task initiation).

The objective of this review was to examine how school-based behavioral and psychosocial interventions support students with chronic conditions in inclusive educational contexts and to analyze their broader symbolic and institutional implications.

### 2.3. Search Strategy

A comprehensive search was conducted across five electronic platforms (databases), PubMed, ERIC, PsycINFO, Scopus, and Web of Science, covering the literature published between January 2010 and April 2025. Search terms were derived from controlled vocabulary, e.g., MeSH and free-text terms, grouped under the PECO framework.

- Population = (children OR adolescents OR “school-aged”);
- Exposure = (“behavioral intervention” OR “psychosocial intervention” OR “self-management” OR “health promotion” OR “coping strategies”);
- Context = (“school-based” OR “inclusive education” OR “classroom-based”);
- Condition = (“chronic illness” OR “chronic disease” OR asthma OR diabetes OR epilepsy OR ADHD OR autism OR “chronic pain”).

The core Boolean syntax used was (school-based OR “inclusive education” OR “classroom-based”) AND (“behavioral intervention” OR “psychosocial intervention” OR “self-management” OR “health promotion” OR “coping strategies”) AND (“chronic illness” OR “chronic disease” OR asthma OR diabetes OR epilepsy OR ADHD OR autism OR “chronic pain”) AND (children OR adolescents OR “school-aged”)

Database-specific adaptations were applied, e.g., [tiab] in PubMed, subject descriptors in ERIC. Truncation symbols, e.g., educat* and filters for peer-reviewed journal articles, were employed. The full search strategies for each database are provided in Supplementary S1.

### 2.4. Inclusion and Exclusion Criteria

Inclusion criteria are as follows:

- Participants: Children and adolescents aged 5–18 with diagnosed chronic health conditions (physical or neurodevelopmental);
- Interventions: Behavioral, psychosocial, or educational programs delivered in school/inclusive settings;
- Outcomes: Reported quantitative or mixed method results in at least one of the following: symptom management, psychosocial wellbeing, academic participation;
- Study designs: RCTs, quasi-experimental studies, or pre/post-intervention studies;
- Language: English;
- Publication type: Peer-reviewed empirical articles (2010–2025).
- Exclusion criteria are as follows:
- Interventions focused exclusively on pharmacological or clinical treatment outside of school settings;
- Studies conducted solely in hospitals or clinical environments;
- Review articles, editorials, commentaries, conference abstracts, dissertations;
- Qualitative-only studies without reportable outcome data.

Although qualitative research was not excluded a priori, no such studies met the inclusion criteria with sufficient outcome data.

### 2.5. Study Selection Process

All records were imported into Rayyan for screening and deduplication. After removing 50 duplicates, 370 records remained for initial screening.

- Phase 1: Titles and abstracts were independently screened by two reviewers (Author 1 and Author 2).
- Phase 2: Fifty full-text articles were retrieved and assessed using a standardized eligibility rubric.
- Discrepancies were resolved through discussion; a third reviewer (Author 3) was consulted when needed.
- Final inclusion of 28 studies that met all criteria.

**Figure 1:**
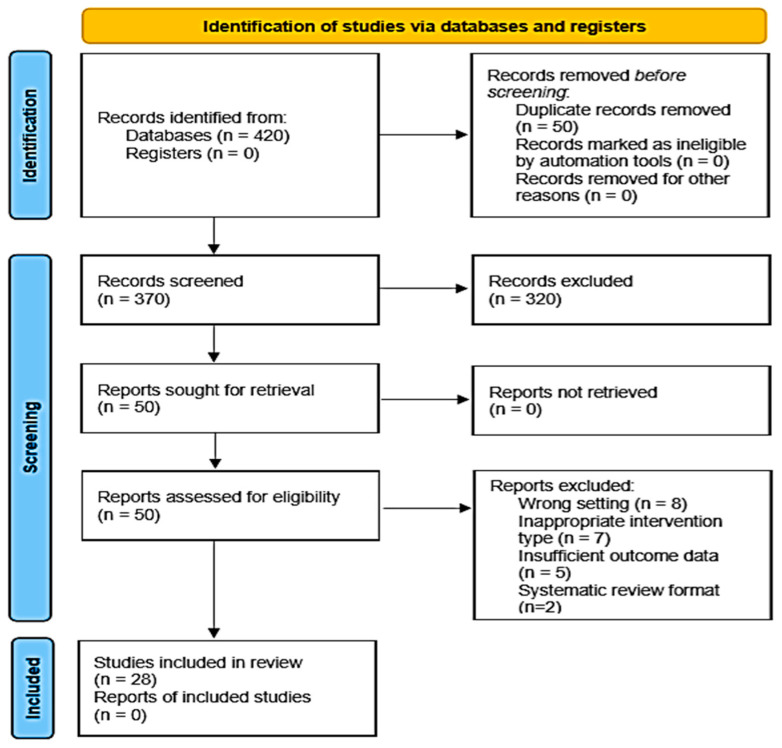
PRISMA 2020 flow diagram illustrating the systematic search and selection process of included studies. The term “databases” is used for the five electronic platforms searched (PubMed, ERIC, PsycINFO, Scopus, Web of Science), and the correct number of records (n = 420) is shown.

The PRISMA 2020 flow diagram (Figure 1) illustrates the selection process. The diagram was constructed using the PRISMA template.

### 2.6. Data Extraction and Management

A structured data extraction template was developed in Excel, including the following:

- Study metadata (author, year, country);
- Population characteristics and sample size;
- Condition focus, e.g., asthma, ADHD;
- Intervention type, e.g., peer-led, digital;
- Study design and comparator, where applicable;
- Outcome domains and key results.

Intervention characteristics were coded based on the Template for Intervention Description and Replication (TIDieR) checklist (Hoffmann et al., 2014 [14]).

Two reviewers independently extracted data; consensus was reached via iterative discussion. No automation tools were used. Extracted data were aligned with the research questions for comprehensive coverage of intervention types, measured outcomes, and the educational or institutional implications.

### 2.7. Quality Appraisal

Study quality was assessed using validated critical appraisal tools:

- RCTs (n = 16) were evaluated using the Cochrane Risk of Bias 2 (RoB 2) tool.
- Non-randomized or quasi-experimental studies (n = 12) were assessed using the Joanna Briggs Institute (JBI) Checklist for quasi-experimental studies.

Two reviewers independently conducted the assessments; disagreements were resolved by a third reviewer. Quality ratings were incorporated into the interpretation of results but not used as exclusion criteria. A detailed summary of study-level quality assessments is presented in Supplementary S2.

### 2.8. Data Synthesis

Given the heterogeneity of interventions, outcome types, and study designs, a meta-analysis was not feasible. Instead, we employed a narrative and thematic synthesis method, following PRISMA 2020 guidance for qualitative integration. Studies were grouped by the following:

- Target condition, e.g., asthma, diabetes, ADHD;
- Intervention modality, e.g., digital tools, teacher training, peer-led programs.

Qualitative data were imported into NVivo 14 software to support inductive coding and thematic development. Themes were derived through interpretive coding and refined via iterative comparison. Two researchers independently coded the data and resolved discrepancies through discussion. Three meta-themes structured the results:

1. Integration of care into school routines;
2. Redistribution of responsibility across school actors;
3. Reconfiguration of student subjectivity and belonging.

These themes informed the structure of Section 3 (Results) and supported the critical analysis in Section 4 (Discussion). They also correspond to the review’s three sub-questions: the types of interventions implemented, their health/academic/psychosocial outcomes, and their implications for inclusive educational practices.

## 3. Results

### 3.1. Overview and Thematic Structure

The 28 studies included in this review were analyzed narratively and thematically, revealing three cross-cutting patterns on chronic conditions, intervention types, and institutional contexts. First, many interventions integrated care practices directly into the spatial and temporal routines of schooling, e.g., through scheduled inhaler use, classroom-based coping strategies, or peer-led support. Second, several programs facilitated a redistribution of care responsibilities, engaging educators, school nurses, students, and parents in collaborative frameworks of health promotion and inclusion. Third, a subset of interventions operated at a symbolic and pedagogical level, challenging established assumptions about ability and reframing chronic illness as a generative difference rather than a deficit to be managed. These themes inform the synthesis below and structure the findings by target condition and intervention approach.

The 28 studies reviewed reflect a multidimensional archive of how schools respond to chronic illness within diverse educational systems. Published between 2010 and 2025, the studies focus on a range of national contexts, including the United States, Canada, the United Kingdom, Jordan, India, and Mexico, indicating a global effort to reconcile chronic care needs with the spatial and temporal constraints of schooling. Methodologically, the studies include randomized controlled trials (n = 16) and quasi-experimental or pre-post designs (n = 12). Furthermore, clinically, they address the following:

- Asthma (n = 12).
- ADHD (n = 6).
- Diabetes (n = 3).
- Epilepsy (n = 2).
- Autism Spectrum Disorder (n = 2).
- Other chronic conditions, including food allergies and cancer survivorship (n = 3).

An overview of intervention categories by chronic condition and implementation strategy is summarized in Table 1. A detailed description of each study’s setting, design, intervention type, outcomes, and findings is presented in Table 2, Table 3, Table 4 and Table 5. 

#### Alignment with Research Questions

The following results are organized thematically in response to the three research sub-questions posed in the introduction.

1. What behavioral or psychosocial interventions have been implemented in school settings to support students with chronic health conditions?
2. What health, academic, and psychosocial outcomes have these interventions produced?
3. How do these interventions reflect or resist prevailing educational assumptions about ability, participation, and normativity?

Each subsection below corresponds to specific condition-focused interventions, e.g., asthma, ADHD, epilepsy, and synthesizes their design, outcomes, and institutional implications.

### 3.2. Asthma and School-Based Care

Asthma-focused interventions were the most frequently represented in the dataset, with ten studies providing structured models for integrating asthma management into school environments. While varied in design, from nurse-supervised therapy to peer-led education, these interventions shared a commitment to integrating care into school life. This subsection responds to the review’s first two sub-questions, identifying asthma-specific behavioral and psychosocial interventions (RQ1) and documenting their health, academic, and psychosocial outcomes (RQ2). It also contributes to the third sub-question (RQ3) by illustrating how such programs disrupt normative educational routines and promote more inclusive models of care.

Several studies emphasized nurse-led models as mechanisms of routine normalization and health stabilization. For example, Trivedi et al. [21] reported that a school nurse-supervised asthma program, which incorporated daily administration of inhaled corticosteroids and regular symptom monitoring, resulted in marked reductions in emergency department visits and improved medication adherence among children with persistent asthma, highlighting how structured care routines within schools can scaffold attendance and ongoing student engagement. In addition, Harris et al. [13] implemented a theatre-based self-management intervention in schools, which promoted asthma awareness and self-care skills among students through participatory educational workshops. This approach expanded school health interventions beyond clinical routines, nurturing student engagement and agency in managing chronic illness within the educational environment. Accordingly, Szefler et al. [23] implemented a care coordination model that bridged school and clinical care through comprehensive asthma management protocols delivered by school nurses and care teams, resulting in improved adherence to asthma action plans and fewer missed school days. Moreover, Halterman et al.’s [16] School-Based Telemedicine Enhanced Asthma Management (SB-TEAM) program integrated digital assessments with directly observed therapy, achieving great reductions in symptom days and urgent healthcare use. These models reframed school as a site of chronic care infrastructure in under-resourced communities.

Beyond clinical supervision, several programs foregrounded student agency and peer learning. Al-Sheyab et al. [17] deployed a peer-led asthma education model in Jordanian secondary schools, which enhanced asthma knowledge and smoking resistance. This aligns with Cicutto et al. [22], whose Canadian intervention, led by public health nurses, improved inhaler technique and attendance through cooperative classroom engagement. McGhan et al. [42] contributed with the Roaring Adventures of Puff (RAP) program, which improved symptom control and activity participation, featuring the relational dimension of student-centered care.

Recent studies have introduced culturally responsive and embodied pedagogies. Kocaaslan et al. [15] demonstrated that a simple animated video on asthma self-management enhanced health knowledge and inhaler skills among children. Chadi et al. [43], conducted a pilot randomized trial comparing in-person and eHealth mindfulness-based stress reduction (MBSR) interventions for adolescents with chronic illnesses, demonstrating that both formats were feasible and led to improvements in psychosocial outcomes, including reduced stress and enhanced mood. Furthermore, McGovern et al. [20]’s breathing retraining and cognitive-behavioral program yielded improved coping strategies and quality of life. Clark et al. [19] evaluated two school-based asthma interventions for preteen students, finding that while neither program changed asthma symptoms or quality of life, both led to improvements in academic performance and self-regulation over a 24-month period. Across these asthma interventions, school time is less fixed and normative, reshaped to accommodate chronic needs. These studies reposition school as a context where health and education are co-produced, care labor is redistributed, and illness is addressed through structural responsiveness and symbolic inclusion. A detailed summary of asthma-focused interventions is presented in Table 2.

**Table 2:** TIDieR summary of asthma interventions.

#	Study (Author, Year)	Country	Condition	Who Delivered	What (Materials)	How (Mode)	Where	When and How Much	Tailoring	Fidelity	Outcomes
1	Kocaaslan et al. [15]	Türkiye	Asthma	Clinician/educator	Animated asthma video	Individual viewing	School/clinic	1 session + booster	Age/language tailored	Checklist follow-up	* ↑ Inhaler technique, ↑ knowledge
2	Halterman et al. [16]	USA	Asthma	Nurse + clinician	** DOT + telemedicine	In-person + telehealth	School	Daily + 3/year	Individualized plans	Visit adherence logs	↓ Symptoms, ↑ adherence
3	Al-Sheyab et al. [17]	Jordan	Asthma	Peer leaders	Peer education module	Peer-led groups	School	Weekly × 3	Culturally adapted	Participation logs	↑ Awareness, ↑ smoking resistance
4	Kawafha & Tawalbeh [18]	Jordan	Asthma	Nurse educator	7-topic training	Teacher workshops	Primary schools	3 sessions	Educator-focused	Pre/post AGKQA	↑ Knowledge, ↑ attitudes
5	Clark et al. [19]	USA	Asthma	Nurses and educators	Self-management toolkit	Group + 1:1	Urban schools	8 weeks	Plans individualized	Logs, attendance	↑ Self-efficacy, ↓ visits
6	McGovern et al. [20]	USA	Asthma	Mental health staff	CBT + breathing exercises	Group sessions	School	6 weekly sessions	Anxiety adapted	Homework logs	↓ Anxiety, ↑ QoL
7	Trivedi et al. [21]	USA	Asthma	School nurses	Inhaled corticosteroids, asthma action plans, medication logs	Nurse-supervised medication administration, family-physician coordination	Schools (Massachusetts)	Daily during school year (pre/post 1 year)	Targeted to students with poor adherence	Compliance logs, communication checks	↓ ED visits, ↓ hospitalizations, ↓ rescue med use, ↑ adherence
8	Cicutto et al. [22]	Canada	Asthma	Public health nurses	Asthma education toolkit	Classroom group	Public schools	4 sessions/4 months	Language/age adapted	Pre/post surveys	↑ Inhaler use, ↑ attendance
9	Szefler et al. [23]	USA	Asthma	School nurses+ care teams	BBAC protocol: asthma education, risk assessment, care plans, case management, linkage to healthcare providers	Group and individual sessions, case management	Schools	Academic year (routine school care)	Adapted to district and student risk levels	Implementation monitoring, care plan documentation	↑ Asthma control, ↑ care plan completion, ↓ absenteeism, ↓ emergency visits
10	Alreshidi et al. [27]	Saudi Arabia	Asthma	School nurses	Asthmahealth education program	Group sessions	Schools	Multiple sessions (2 intervention, 2 control)	Standardized program, no reported tailoring	Pre/post questionnaires	↑ Knowledge, ↑ quality of life, ↓ absenteeism, no change in attitude or anxiety
11	Eakin et al. [28]	USA	Asthma	Educators + staff	iPad videos + handouts	Home + workshop	Head Start centers	4 visits + follow-up	Trigger-based	App logs	↑ Asthma control, ↓ ER use
12	Harris et al. [13]	UK	Asthma	Theatre educators + asthma educators	Theatre-based education, asthma self-management toolkit	Theatre performance + small group self-management workshops	Secondary schools	One theatre session, one follow-up workshop, 6 months follow-up	Culturally/contextually adapted for schools	Attendance records/participation monitoring	↑ Asthma knowledge, ↑ perception, improved self-management, high feasibility/acceptability

* ↑ = improvement/increase; ↓ = reduction/decrease. ** CBT = cognitive behavioral therapy; DOT = directly observed therapy; QoL = quality of life; AGKQA = Asthma General Knowledge Questionnaire for Adults; ER = emergency room.

### 3.3. ADHD and the Reorganization of Classroom Participation

Interventions targeting students with attention-deficit/hyperactivity disorder (ADHD) accommodate neurodiverse temporalities and executive function profiles. These studies framed ADHD as a behavioral challenge and a difference in how students engage with the structured demands of school life. This section addresses all three research sub-questions, as it identifies ADHD-focused behavioral and psychosocial interventions (RQ1), examines their reported academic, behavioral, and psychosocial outcomes (RQ2), and explores how these interventions challenge normative school practices by redefining classroom participation and institutional responsibility (RQ3).

A central strategy across studies involved behavioral scaffolding through feedback systems and organizational supports. Sibley et al. [35] evaluated a community-based behavioral therapy program for adolescents with ADHD, showing reductions in oppositional behavior and comorbid symptoms alongside gains in attention and organization. This daily feedback structure acted as a temporal scaffold, segmenting the school day into moments of recognition, regulation, and support.

Additionally, Langberg et al. [36] advanced this model with the HOPS program (Homework, Organization, and Planning Skills), which improved homework productivity and task management among middle school students. These interventions did not aim to normalize behavior, but to align institutional expectations with neurocognitive diversity, expanding what ‘participation’ looks like.

The setting of intervention delivery also shaped outcomes. Evans et al. [32] compared after-school skills training with in-school mentoring, finding the former more effective in improving attention and academic behaviors. This raises important questions about the temporal and spatial constraints of school-based implementation and the need for adaptive program design. Other studies adopted multi-agent, relational models. Sibley et al. [31] implemented a technology-enhanced, multi-tiered intervention combining parent, teacher, and student training. Grounded in the cultural context of participating communities, the program achieved reductions in ADHD symptoms and oppositional behavior. This approach highlights the value of systemic coordination and ecologies of care over individual behavioral management.

Teacher-focused interventions by DuPaul et al. [33] and Pfiffner et al. [34] emphasized professional development and communication strategies. While both studies showed positive shifts in teacher knowledge and school-home collaboration, they highlighted the structural limitations educators face when institutional supports are lacking. Taken together, ADHD interventions demonstrate efficacy when they redistribute regulatory labor, equipping students with adaptive tools while compelling schools to assume responsibility for temporal and behavioral flexibility. Details of the ADHD interventions are summarized in Table 3.

**Table 3:** TIDieR summary of ADHD interventions.

#	Study (Author, Year)	Country	Condition	Who Delivered	What (Materials)	How (Mode)	Where	When and How Much	Tailoring	Fidelity	Outcomes
13	Sibley et al. [31]	Mexico	ADHD	Therapists	Multi-tier behavior therapy	Group delivery	Middle schools	12 weeks	Tiered by severity	Progress logs	* ↓ Symptoms, ↑ organization
14	Evans et al. [32]	USA	ADHD	Teachers and mentors	Skills training vs. mentoring	Group + 1:1	After-school settings	8 sessions	Behaviorally targeted	Attendance sheets	↑ Behavior, ↑ academic outcomes
15	DuPaul et al. [33]	USA	ADHD	Teachers + parents	Consultation + home plan	Collaborative plan	Schools + home	12 weeks	Individualized supports	Observation + reports	↑ Collaboration, ↓ inattention
16	Pfiffner et al. [34]	USA	ADHD	Multidisciplinary team	Collaborative Life Skills	Group + family	Elementary schools	12 weeks	Whole-child focus	Checklists, ratings	↓ Oppositional behavior, ↑ engagement
17	Sibley et al. [35]	USA	ADHD	Clinicians + families	Family-centered ** CBT	Family sessions	Home + school	10 sessions	Adolescent-tailored	Session logs	↑ Time mgmt, ↓ impairment
18	Langberg et al. [36]	USA	ADHD	School psychologists	HOPS exec function training	Small groups	Middle schools	8–10 weeks	Planning-focused	Assignment logs	↑ Homework, ↑ planning

* ↑ indicates increase or improvement; ↓ indicates decrease or reduction. ** CBT = cognitive behavioral therapy; HOPS = homework, organization, and planning skills; mgmt = management.

### 3.4. Responsibility and Readiness in Diabetes and Epilepsy

Interventions targeting diabetes and epilepsy accentuate how schools reconfigure responsibility and institutional readiness in the management of chronic illness. These studies emphasize technical competence and ethical and symbolic shifts in how schools engage with children’s health. This subsection responds to the review’s three guiding sub-questions by (1) identifying school-based interventions for diabetes and epilepsy (RQ1); (2) examining outcomes related to staff preparedness, student autonomy, and stigma reduction (RQ2); and (3) analyzing how such interventions redistribute responsibility and challenge exclusionary assumptions about student risk and competence (RQ3).

In the context of Type 1 diabetes, responsibility is distributed across the child, school personnel, and healthcare providers, reflecting a model of shared care. Al-Daghri et al. [25] implemented an e-learning program for educators in Saudi Arabia, which produced sustained knowledge gains and enhanced staff confidence in diabetes care over a 12-month follow-up. This shift, from uncertainty to professional assurance, demonstrates how training transfers authority and accountability to educational staff in a culturally responsive manner. Furthermore, Smith et al. [24] reported that brief, in-person workshops improved educators’ preparedness and first-response efficacy, suggesting that medical support and pedagogical responsibility are not mutually exclusive. Peery et al. [26] extended this model through a nurse-led diabetes case management program in U.S. schools, which nurtured student self-efficacy and enhanced trust among teachers and families.

Interventions addressing epilepsy confront stigma and misrecognition Alkhotani and Alkhotani [29] demonstrated that a brief health education session for primary school teachers in Saudi Arabia improved their knowledge of seizure first aid. While the intervention focused on teacher knowledge rather than direct peer education, such efforts are crucial first steps toward creating safer and more supportive school environments for students with epilepsy and may contribute to reducing stigma through increased preparedness and understanding among school staff. Eze et al. [30] achieved similar results among pre-service teachers in Nigeria, whose increased knowledge and reduced bias reflected a cultural reorientation toward inclusion. Schools become sites of ethical labor, where readiness and responsibility are enacted through pedagogical relationships and collective care practices. An overview of intervention formats and delivery characteristics is presented in Table 4.

**Table 4:** TIDieR summary for diabetes and epilepsy interventions.

#	Study (Author, Year)	Country	Condition	Who Delivered	What (Materials)	How (Mode)	Where	When and How Much	Tailoring	Fidelity	Outcomes
19	Smith et al. [24]	USA	Diabetes	School staff	Emergency response workshop (curriculum)	In-person training	Schools	1 session	Emergency-focused	Pre-/post-tests	* ↑ Confidence in emergency response ↑ knowledge
20	Al-Daghri et al. [25]	Saudi Arabia	Diabetes	Teachers	Online diabetes course	Online modules	Remote	Self-paced	Cultural adaptation	Completion tracking	↑ Confidence, ↑ care skills
21	Peery et al. [26]	USA	Diabetes	Nurses	Diabetes care management	Individual sessions	Schools + home	Ongoing	Student–family adapted	Logbooks, feedback	↑ Self-efficacy, ↑ parent–teacher trust
22	Alkhotani et al. [29]	Saudi Arabia	Epilepsy	Health educators	Seizure first aid education session	In-person session	Primary schools	Single session	Standardized session; tailoring not described	Pre/post testing	↑ Knowledge of seizure first aid
23	Eze et al. [30]	Nigeria	Epilepsy	Teacher educated	Epilepsy training	Workshop	Teacher colleges	1–2 days	Localized context	Post-training quiz	↓ Bias, ↑ preparedness

* ↑ indicates increase or improvement; ↓ indicates decrease or reduction. All acronyms are explained in the table or in the text.

### 3.5. Interventions Across Autism, Cancer, and Allergies

While clinically diverse, the interventions addressing autism spectrum disorder (ASD), cancer survivorship, and life-threatening allergies converge in their challenge to normative pedagogical structures. These conditions are socially marked, symbolically overdetermined, and complex within school cultures. This subsection addresses the review’s three guiding sub-questions by (1) identifying interventions aimed at neurodevelopmental, immunological, and post-treatment conditions (RQ1); (2) evaluating outcomes such as peer inclusion, emotional recovery, and emergency preparedness (RQ2); and (3) analyzing how these interventions reframe institutional roles, disrupt deficit-based assumptions, and advance inclusive pedagogical practices (RQ3).

In the case of autism, interventions reoriented neurotypical peer responses and adult expectations. Kasari et al. [37] implemented a peer-mediated social skills training program in U.S. elementary schools, which resulted in improved peer connectivity and reduced playground isolation among autistic students. Rather than focusing on behavioral remediation, the intervention challenged dominant deficit models, instead inviting neurotypical peers to understand and adapt to neurodivergent social communication styles. Locke et al. [38], through the Remaking Recess initiative, trained staff to promote inclusive peer interactions during recess, which is overlooked in formal interventions. These practices reframed recess as an intentional social inclusion, not incidental socialization.

The return of childhood cancer survivors to school might obscure the affective and academic challenges of reintegration. Thompson et al. [39] examined re-entry programs that provided structured emotional and educational support, facilitating collaboration between families, teachers, and medical professionals. These interventions reframed survivorship as a process of shared responsibility, where schools are ethically accountable for easing the transition and the complexities of recovery. Relatedly, Douma et al. [41] evaluated an online group psychosocial intervention for Dutch adolescents with a range of chronic illnesses, finding improvements in disease-related coping skills and health-related quality of life, including school and social functioning. By providing a protocolled group setting that nurtures adaptive coping, this intervention demonstrates how psychosocial support is efficiently integrated into the school context and disrupts the isolation that marks chronic illness in adolescence.

In addition, Kourosh et al. [40] explored interventions for food allergies that necessitate vigilance and preparedness. Their study showed that structured staff training in urban U.S. schools improved emergency responsiveness and educator confidence. These interventions decenter medical pathology and instead spotlight how schools perform care, symbolically, spatially, and structurally. Freire [9] mentioned that education is a political act, and in this context, inclusion becomes a form of redistributive justice, where voice, agency, and protection are integrated in the pedagogical order. TIDieR-based details of these diverse interventions are included in Table 5.

**Table 5:** TIDieR summary for autism, cancer, and allergy interventions.

#	Study (Author, Year)	Country	Condition	Who Delivered	What (Materials)	How (Mode)	Where	When and How Much	Tailoring	Fidelity	Outcomes
24	Kasari et al. [37]	USA	Autism	Researchers + staff	Peer social skills training	Group sessions	Mainstream classrooms	8 weeks	Peer-mediated, inclusive	Session logs	* ↓ Isolation, ↑ peer interaction
25	Locke et al. [38]	USA	Autism	School staff	Recess peer facilitation	Playground activities	Elementary playgrounds	Daily recess periods	Informal setting tailored	Staff observation	↑ Inclusion, ↑ social interaction
26	Thompson et al. [39]	USA	Cancer (re-entry)	Educators + counselors	Re-entry support program	Individual + group	Schools	Tailored per student	Emotionally responsive	Participation tracking	↑ Wellbeing, ↑ reintegration outcomes
27	Kourosh et al. [40]	USA	Food Allergy	School staff	Allergy response training	Workshop + simulation	Urban public schools	1 session + drills	Emergency context	Checklists, drills	↑ Staff confidence, ↑ emergency response
28	Douma et al. [41]	Netherlands	Chronic illness	Psychologists/Trained group leaders	Op Koers Online protocol (manualized online psychosocial group program)	Synchronous online group sessions (video chat)	At home (remote/online)	Six weekly sessions (1.5 h each), with follow-up boosters	Not specified (protocol-driven)	Session attendance tracked, standardized delivery	↑ Disease-related coping, ↑ HRQoL (social, school, psychosocial functioning)

* ↑ indicates increase or improvement; ↓ indicates decrease or reduction. All acronyms (e.g., CBT: cognitive behavioral therapy; RCT: randomized controlled trial; ADHD: attention-deficit/hyperactivity disorder) are explained in the text.

### 3.6. From Accommodation to Institutional Restructure

While ostensibly designed to support students with chronic conditions, the interventions reviewed unsettle normative assumptions about where and how care occurs within educational settings. These programs prompt schools to move beyond accommodation and toward institutional reconfiguration, including health-related practices into schooling. This section synthesizes perspectives across conditions and interventions to respond to the review’s three sub-questions: (1) it identifies institutional-level adaptations prompted by diverse behavioral and psychosocial programs (RQ1); (2) it highlights outcomes that transcend symptom management, extending into pedagogical restructuring and collective preparedness (RQ2); and (3) it interrogates how these interventions challenge normative educational assumptions and foreground a relational ethics of inclusion (RQ3).

For instance, Trivedi et al. [21] evaluated a school nurse-supervised asthma program in which daily administration of inhaled corticosteroids and routine symptom monitoring were integrated into the school environment, resulting in reduced emergency department visits and improved medication adherence among children with persistent asthma. In contrast, Harris et al. [13] implemented a theatre-based self-management intervention, emphasizing asthma awareness and self-care skills among students through educational workshops integrated within the school setting. This challenges the traditional bifurcation between health and education, demonstrating that chronic illness care is part of the school’s everyday operations. Moreover, interventions for ADHD, like Langberg et al.’s [36] HOPS program or Sibley et al.’s [31] multi-tiered supports, treat time management, executive function, and organization as pedagogical tasks. These are teachable, supportable skills found within the curriculum.

In epilepsy and life-threatening allergies, the threat of seizure or anaphylaxis shifts the school’s role to an agent of preparedness. Programs by Alkhotani et al. [29] and Kourosh et al. [40] demonstrate that risk, when acknowledged and collectively addressed, becomes a symbolic manifestation of inclusion. The school develops a shared vocabulary of care, beyond medical knowledge that extends to attentiveness and ethical responsibility. These transformations are evident in the relational dynamics and role reconfigurations that interventions initiate. Peer mediators in autism programs (Kasari et al. [37]) take on the roles of social facilitators. Teachers trained in diabetes support (Smith et al. [24]) become co-regulators of bodily care. In care coordination models, according to Szefler et al. [23], school nurses and care teams act as trans-institutional connectors, implementing comprehensive asthma management protocols that bridge classroom realities with clinical expertise and facilitate improved health outcomes for students. 

### 3.7. Quality of Evidence

The methodological foundation of the 28 included studies reflects both the strengths and challenges of conducting health-focused interventions within dynamic school environments. Of these, 18 were randomized controlled trials (RCTs) and 10 were quasi-experimental designs, including pre–post interventions and non-randomized trials. This review was reported in accordance with PRISMA 2020 guidelines to ensure a transparent and comprehensive presentation of methods and findings. However, the assessment of methodological quality was conducted using study-design-specific tools, the Cochrane Risk of Bias 2 (RoB 2) tool for RCTs and the Joanna Briggs Institute (JBI) Checklist for quasi-experimental studies. Most RCTs demonstrated low to moderate risk of bias in randomization, outcome measurement, and participant retention. Common methodological limitations included the following:

- Lack of blinding among educators and implementers;
- Short intervention durations (fewer than three months);
- Limited long-term follow-up data.

These constraints are characteristic of school-based research, where ethical obligations, staff turnover, and policy cycles limit opportunities for extended study designs or double-blinding protocols.

The quasi-experimental studies showed greater variability in quality. While several incorporated robust design features, like baseline equivalence and multi-informant outcome reporting, others were constrained by single-group pre-post designs, modest sample sizes, or the absence of statistical controls. Despite these limitations, these studies offered ecological validity by capturing the real-world dynamics of educational institutions, contributing contextual information to our third research question (RQ3) on institutional norms and symbolic structures. No study was excluded based on methodological quality. Instead, quality appraisals were used to modulate the interpretive weight assigned to individual findings, in relation to intervention effectiveness and generalizability. This approach values internal validity and the capacity of school-based interventions to reshape symbolic, pedagogical, and institutional boundaries. A detailed summary of quality appraisal results is presented in Supplementary S2 (Quality appraisal summary of included studies).

Risk of bias and methodological quality assessments are summarized in Table 6 below. Of the 18 randomized controlled trials (RCTs), most were rated as low risk or some concerns on the RoB 2 tool, mainly due to the practical challenges of blinding and short intervention durations typical in educational settings. Of the 10 quasi-experimental studies, several were rated as high risk of bias on the JBI Checklist, often due to the use of single-group pre/post designs, small sample sizes, or lack of statistical controls. However, some quasi-experimental studies included baseline equivalence or multi-informant outcome reporting, enhancing their ecological validity. The full results of these quality appraisals, including notes on potential sources of bias for each study, are detailed in Supplementary S2.

Interpretation and implications:

This appraisal indicates that while most RCTs in the review provide a reasonable degree of internal validity, shared methodological limitations in blinding and follow-up temper our confidence in reported effects. The higher risk of bias among quasi-experimental studies warrants a more cautious interpretation of their findings regarding generalizability. These considerations are reflected in our synthesis and discussion, where the strength of evidence is weighed accordingly for each intervention model.

### 3.8. Academic, Psychological, and Health Domains of Impact

This section addresses Research Question 2, which examined the health, academic, and psychosocial outcomes of school-based behavioral and psychosocial interventions for students with chronic conditions. Across the 28 studies reviewed, on asthma, autism, diabetes, epilepsy, cancer, ADHD, and food allergies, interventions demonstrated cross-cutting impacts across these three outcome domains. These findings resist narrow evaluations of effectiveness. Instead, they invite a broader conceptualization of inclusion as a multidimensional institutional endeavor. Successful outcomes required more than clinical or behavioral efficacy as they relied on access to consistent care, empathetic relationships, institutional flexibility, and attentiveness from the school system.

Health outcomes were foundational in interventions targeting asthma, diabetes, and allergies. For example, Trivedi et al. [21] demonstrated that a school nurse-supervised asthma management program, which incorporated daily administration of inhaled corticosteroids and routine symptom monitoring, led to reductions in emergency department visits, improved medication adherence, and decreased healthcare utilization among children with persistent asthma. Similarly, Szefler et al. [23] and Peery et al. [26] reported reductions in symptom severity, improved medication adherence, and decreased school absenteeism. These effects emerged through systemic coordination, involving trained personnel, reliable care routines, and strong communication between schools and health providers. Health, in this context, emerges as a construct that is co-produced within the institutional setting, in school life.

Academic outcomes were prominent in ADHD and cancer-related interventions. Langberg et al. [36] and Evans et al. [32] reported gains in executive functioning, classroom engagement, and task completion, reflecting interventions that scaffold time management and align instructional practices with neurodiverse learning needs. In the context of cancer survivorship, Thompson et al. [39] emphasized the process of academic re-entry, supporting curricular reintegration, identity reconstruction and peer connection, thereby reframing learning.

Psychosocial outcomes extended beyond traditional health or academic markers. Interventions suggested by Alkhotani et al. [29], Locke et al. [38], and Kourosh et al. [40] increased peer empathy, reduced stigma, and improved emotional resilience. Douma et al. [41] equally found that participation in a structured, online psychosocial group intervention improved coping skills and health-related quality of life, including school and social functioning, among Dutch adolescents with chronic illnesses. These findings are supported by Wahlström et al. [4], who demonstrated that supportive psychosocial working conditions within schools significantly contribute to adolescents’ overall life satisfaction. These outcomes reframe schools as contexts of symbolic recognition, where vulnerability is a condition of ethical and educational engagement. Inclusion, in this framework, is a matter of institutional reflexivity and relational care. This synthesis aligns with PRISMA 2020 guidelines for narrative and thematic integration, showing how outcomes manifest across diverse interventions and chronic conditions.

## 4. Discussion

This discussion addresses the main research question that guided this review: “How do school-based behavioral and psychosocial interventions support children and adolescents with chronic health conditions in inclusive educational settings, across health, academic, and psychosocial domains?” Based on the synthesized findings and thematic analysis, we examine how these interventions reshape institutional norms, pedagogical practices, and symbolic logics within schooling. Each subsection below responds to the review’s sub-questions, interpreting the broader implications of intervention types, outcomes, and ideological assumptions.

### 4.1. How Chronic Illness Redefines Schooling

This discussion responds to the main research question guiding the review, “How do school-based behavioral and psychosocial interventions support children and adolescents with chronic health conditions in inclusive educational settings, across health, academic, and psychosocial domains?” Drawing from the narrative synthesis of 28 studies, this section examines how chronic illness reconfigures schooling, prompting shifts in pedagogy, care, and institutional responsibility. The interventions evaluated in this review, ranging from asthma education programs and diabetes care coordination to ADHD executive functioning support and epilepsy stigma reduction, differed in clinical focus, delivery method, and institutional integration. This heterogeneity necessitated a narrative synthesis, in accordance with PRISMA 2020 guidelines, to identify cross-study patterns with contextual specificity. What emerges is a plethora of educationally integrated responses to chronic illness. These include nurse-led protocols for medication management, peer-led awareness campaigns, digital self-monitoring tools, and multi-agent collaboration models that involve families, educators, and external providers [6,8,13,33]. Beyond clinical add-ons, such interventions interrupt the traditional perspective of chronic illness as an external disruption, with chronicity as a pervasive and structuring aspect of school life.

This perspective is echoed in Daly et al.’s [1] findings, which emphasize that many educators feel ill-equipped to support students with chronic illnesses, leading to inconsistent accommodations and heightened risk for marginalization. Additionally, Sentenac et al. [2], using data from 19 European countries, show that children with chronic health conditions report lower school satisfaction, highlighting the need to reconceive school environments and their health responses. From this angle, schools are negotiated contexts of biopolitical care, where responsibilities, identities, and institutional norms are restructured in response to chronic illness. Interventions, like inhaler routines for asthma, seizure response plans, and daily blood glucose monitoring, represent isolated health practices as pedagogical acts that integrate care into school life, redistributing medical knowledge and responsibility among educators, students, and families [5,35].

These interventions position inclusion as a normative feature of educational planning and culture. Runions et al. [3] and Yu et al. [6] argue that schools that adopt a whole-child, multi-component approach to inclusion might move beyond individualized solutions to achieve systemic transformation, redesigning time, curriculum, peer engagement, and institutional routines to reflect student health diversity. Thus, inclusion, in this reframed context, is less about physical presence or academic access and more about structural adaptability, relational care, and ethical responsiveness with schools co-constructing health, identity, and belonging.

### 4.2. Making Room for Illness in the Classroom

This section responds to the first sub-question of the review, “What types of school-based behavioral and psychosocial interventions have been implemented to support students with chronic health conditions?” The studies reviewed reveal positive outcomes for students with asthma, diabetes, ADHD, and epilepsy, as interventions resulted in reduced symptom days, notably for asthma and respiratory conditions, with improved attendance and academic engagement [15,17,20]. Improvements in executive functioning, self-efficacy, and task-related behavior were also reported in programs targeting ADHD and behavioral challenges [31]. Moreover, studies that integrated peer involvement or educator training showed enhanced psychosocial outcomes, like, reduced stigma, improved peer relationships, and better emotional adaptation [7,11,25]. These outcomes reflect a diverse range of intervention types, e.g., nurse-led health management, teacher-mediated classroom strategies, digital self-monitoring tools, peer-led education, and multi-agent training models related to school, family, and clinical actors. These interventions served as levers of institutional change, including care practices into schools’ pedagogical routines.

Additionally, asthma education programs suggested by Liptzin et al. [44], Eakin et al. [28], and Szefler et al. [23] reposition inhaler use as a normalized school activity, turning clinical management into a daily ritual interwoven with learning schedules. In Szefler et al.’s model, comprehensive protocols delivered by school nurses and care teams further include asthma care into school day, supporting health and educational engagement. In these cases, the classroom becomes a therapeutic and instructional context, where care is enacted by students, teachers, and health professionals. Similar outcomes have been observed internationally. For example, nurse-led asthma education interventions in Jordan and Saudi Arabia have demonstrated improvements in children’s knowledge, attitudes, and quality of life, further supporting the integration of asthma management within the educational environment (Kawafha & Tawalbeh [18]; Alreshidi et al. [27]). Furthermore, diabetes management programs [9,12] empower educators and school staff with the knowledge to provide flexible, real-time support, enabling children to participate in classroom activities without compromising safety or learning. These interventions stress recognition rather than remediation, as they shift from individual adaptation to collective reconfiguration, with schools becoming structurally responsive to chronic illness, accommodating and anticipating health-related needs. Sentenac et al. [2] show that simply placing chronically ill students in mainstream classrooms without sufficient adaptation might lead to negative school experiences. Thus, behavioral and psychosocial interventions reconfigure the temporal, social, and spatial dimensions of schooling to make room for illness as a legitimate, expected, and accommodated part of student life. Such a shift reflects inclusive education as dynamic, relational, and ethically grounded, as called for by Yu et al. [6] and Runions et al. [3]. Consistent with the review’s conceptual framework, this section interprets intervention types through a socio-educational perspective with health, pedagogy, and relational belonging, co-constructing the meaning and practice of inclusion beyond narrow clinical outcomes.

### 4.3. Rethinking What Inclusion Means

This section addresses the third sub-question of the review, “How do school-based behavioral and psychosocial interventions reflect or resist prevailing educational assumptions about ability, participation, and normativity?” The reviewed interventions challenge the educational ideal of the autonomous, self-regulating learner. By including care practices into school routines, through inhaler schedules, flexible testing protocols, peer support strategies, or teacher-led emotional scaffolding, these programs destabilize normative assumptions about time, performance, and independence. Rather than enforcing conformity to neurotypical or able-bodied standards, they acknowledge the legitimacy of interdependence, episodic need, and difference as integral features of the school experience. Sentenac et al. [2] illustrate that, when chronically ill students are placed in mainstream settings without adequate support, their school experiences deteriorate, reinforcing exclusion under the pretense of inclusion, highlighting the insufficiency of inclusion as placement. Rather than a surface-level adjustment, inclusion needs to redefine what schools represent, whom they serve, and how they understand participation. As Foucault [8] theorized, schools are institutions of discipline and normalization, shaping bodies and behaviors through practices of surveillance and control. Genuine inclusion therefore requires the integration of students with chronic illness into existing structures, with a reimagining of those structures themselves, so that difference is valued as a source of collective change.

These interventions move in that direction. They invite educators to decenter normativity by recognizing chronic illness as a structurally anticipated aspect of schooling. According to Slee [10], this shift implies that schools undergo a deeper change in their symbolic and material structures. In such a reorientation, illness prompts educators to build systems of relational engagement, shared accountability, and anticipatory care. The logic of care advanced by Noddings [11] adds a theoretical perspective here. When care is positioned as a structuring principle of educational design, effective inclusion is about reconfiguring schools. This principle is embodied in interventions such as Op Koers Online, evaluated by Douma et al. [41], where group-based psychosocial support for adolescents with chronic illnesses reframed coping, participation, and school belonging as collaborative and relational processes, rather than individual deficits or exceptions. This perspective is reflected in the broader evidence synthesized in this review. Lin et al. [7] emphasize that schools with positive psychosocial climates, characterized by peer support, teacher responsiveness, and reduced academic pressure, better serve the mental health of chronically ill youth. Daly et al. [1] advocate for systemic partnerships and training for schools to shift from reactive accommodations to proactive inclusion. Yu et al. [6] argue that mental health-promoting educational models require attention to curriculum, relationships, and extracurricular life, rather than simply a clinical response.

### 4.4. What Is Missing and Why It Matters

This final section addresses the central research question by examining the structural and epistemological limits of current school-based behavioral and psychosocial interventions for students with chronic illness. It explores whose perspectives are privileged, which regions are overrepresented in the evidence, and how these patterns of representation influence what is recognized and what remains overlooked in contemporary inclusion practices. Most of the studies included in this review were conducted in high-income Western contexts, e.g., the United States, Canada, and the United Kingdom. This concentration reflects infrastructural capacity and research funding availability, but it raises crucial concerns about the transferability, equity, and cultural responsiveness of the findings. Mejía-Rodríguez and Kyriakides [5] emphasize that system-level factors, including national policy, funding, and stratification, substantially shape educational outcomes. Yet, the underrepresentation of the Global South in this evidence constrains our ability to assess how chronic illness is experienced and addressed in diverse educational settings. In settings with weaker school–health systems or different cultural conceptions of illness, the models reviewed may be inapplicable or even counterproductive.

Equally significant is the systematic absence of children’s own voices across much of the literature. While teachers, nurses, and caregivers are consulted, students’ perspectives on their emotional, relational, and embodied experiences are mediated through adult proxies or reduced to quantitative measures of attendance or symptom control. This dynamic is evident in studies with strong health outcomes, e.g., Cicutto et al. [22], where students are seen as passive recipients of institutional care rather than active co-constructors of educational and health interventions.

This absence is ethical and epistemological. Lundy and McEvoy [12] argue that inclusive practice needs to identify children as rights-holders and epistemic agents. When their experiences are marginalized, interventions risk reproducing adult-centric models of schooling and care. Chronic illness, with its episodic, fluctuating, and subjective dimensions, is prone to misrepresentation if youth lack empowerment in articulating their needs and preferences.

Methodologically, several challenges are found in the reviewed studies. While randomized controlled trials (RCTs) offer internal validity and statistical precision, they feature short intervention durations and fail to capture long-term or relational effects. Moreover, the rigid structure of many RCTs may inadvertently exclude contextual factors, e.g., classroom dynamics, peer interactions, or cultural meaning-making, to better understand inclusion. Quasi-experimental and observational studies, although sometimes more ecologically valid, might lack multi-informant triangulation, limiting the interpretive depth to understand intervention impact from multiple stakeholder viewpoints. Moreover, these methodological gaps imply that schools are oriented toward standardization, efficiency, and measurable outcomes. Yet inclusion, as understood in this review, requires contextual, subjective meaning and affective labor, as qualities that are missed from dominant research paradigms. In accordance with PRISMA 2020 guidelines, this review identifies limitations as integral perspectives into how knowledge is constructed, constrained, and is actionable in school-based intervention research.

This review has several limitations. First, the included studies are geographically concentrated in high-income countries, limiting transferability. Second, the prevalence of randomized controlled trials, while offering internal validity, reduces the capacity to identify long-term, relational, and context-sensitive outcomes. Third, the perspectives of students were largely absent across studies, raising epistemological concerns regarding whose experiences are legitimized. Although many of the included studies did not fully report key intervention features such as fidelity, tailoring, or delivery mode, we addressed this by using the TIDieR (Template for Intervention Description and Replication) checklist (Hoffmann et al., 2014) [14] to systematically extract and summarize available intervention characteristics in structured tables. This strengthens transparency, enhances comparability, and supports future implementation.

An additional limitation is that the protocol for this systematic review was not prospectively registered in a public repository (such as PROSPERO or INPLASY), primarily due to time constraints and overlapping institutional ethics review processes. However, registration was not required, as the review did not involve primary data collection from human or animal subjects. Although all methodological steps were predefined and documented internally, the absence of formal registration may increase the risk of reporting bias, as noted in the PRISMA 2020 guidelines. Nonetheless, a key strength of this review lies in its narrative synthesis approach, which allowed for interpretive depth and theoretical framing across diverse evidence, contributing to the understanding of inclusion as an evolving educational practice rather than a fixed outcome.

### 4.5. Risk, Care, and Change

This section synthesizes findings in relation to the third sub-question of how school-based interventions both support individual students and challenge prevailing educational norms. The review found that behavioral and psychosocial interventions do more than alleviate symptoms or advance self-management as they instantiate a shift in how risk, care, and change are understood within school systems. Programs such as asthma education initiatives [12,16], epilepsy stigma reduction campaigns [37], and ADHD-focused executive function coaching [15,20] consistently showed improvements across health and academic metrics, reducing symptom days, increasing attendance, boosting self-efficacy, and nurturing peer relationships. However, these outcomes show how they destabilize traditional school tenets premised on standardization, independence, and meritocratic performance.

Rather than framing risk as a liability to be excluded or minimized, these interventions reframe it as a call for preparedness, empathy, and collective response. Care is no longer relegated to special education or school nurses, but is distributed across relationships, settings, and routines, taken up by teachers, peers, administrators, and digital infrastructures. This broadening of responsibility reflects a movement toward what Noddings describes as an “ethic of care” [11], where education is aligned with relational attentiveness rather than individual achievements. Programs that normalize daily inhaler use, train teachers in insulin management, or build peer support groups represent infrastructural shifts in how schools relate to bodily diversity, modeling an inclusive paradigm where institutional change is a core mechanism of effective intervention.

According to PRISMA 2020 recommendations, this section identifies key implications for policy and practice. First, schools need support to integrate care within their structures through sustained funding, intersectoral collaboration, and teacher training. Second, policy frameworks need to recognize chronic illness as an educational issue and design inclusive approaches accordingly. Third, future interventions need to be co-developed with students to ensure that their experiences shape the definition of inclusive and responsive schooling. These strengths and limitations, along with the implications identified, are consistent with the PRISMA 2020 emphasis on methodological transparency and knowledge translation.

## 5. Conclusions

This systematic review explored the central research question, “How do school-based behavioral and psychosocial interventions support children and adolescents with chronic health conditions in inclusive educational settings, across health, academic, and psychosocial domains?” Through a narrative synthesis of 28 peer-reviewed studies (2010–2025), this review provided an integrated analysis of intervention types, outcomes, and institutional implications, adhering to PRISMA 2020 guidelines. The findings challenge prevailing assumptions that frame inclusion as the simple integration of students into pre-existing educational norms. Instead, the evidence suggests that chronic illness reconfigures the pedagogical and structural conditions of schooling, urging for a more relational and responsive model of inclusion.

In response to Sub-question 1, the review identified a diverse array of interventions, including the following:

- Asthma management programs led by school nurses or delivered digitally;
- Peer-led education for epilepsy, autism, or cancer reintegration;
- Executive function training for students with ADHD;
- Teacher training on chronic illness care, e.g., diabetes management;
- Whole-school psychosocial supports, included in daily routines.

These interventions addressed both visible and invisible dimensions of chronic illness and were delivered through multiple modalities, e.g., face-to-face, digital, or hybrid, across primary and secondary school contexts.

In addressing Sub-question 2, the review synthesized consistent outcome trends, including improvements in symptom control, reduced absenteeism, increased medication adherence, advanced task management, better psychosocial adjustment, and strengthened peer and educator relationships. Several interventions reported reductions in stigma and heightened student agency, reflecting a multidimensional impact that goes beyond clinical metrics.

In response to Sub-question 3, the analysis revealed how these interventions challenge dominant educational assumptions about time, productivity, and ability. Rather than requiring students to adapt to rigid institutional norms, effective interventions prompted schools to evolve, redistributing care, recognizing interdependence, and foregrounding relational ethics in daily practice.

However, there are several gaps, as most studies were situated in high-income countries, with limited representation from culturally and economically diverse contexts. Additionally, the absence of children’s voices across many studies reflects an adult-centric bias in both design and evaluation. Methodologically, few studies included longitudinal follow-up or triangulated perspectives from multiple stakeholders, e.g., teachers, parents, and students, constraining the depth of impact assessment.

Thus, future research needs to perform the following:

- Prioritize participatory approaches that center students’ experiences;
- Invest in long-term evaluation to assess sustained outcomes;
- Expand geographically to include underrepresented global contexts in the Global South.

In conclusion, this review demonstrates that when care is rooted within the everyday practices, relationships, and institutional structures of schools, chronic illness provides a generative perspective for rethinking educational inclusion. Rather than treating students with chronic conditions as peripheral cases, their experiences reveal the structural limitations of normative schooling, accentuating the need for sustained, system-level reconfiguration. Pivotal to this reorientation is the recognition of student voices as influential to the design of effective and fair interventions. Inclusion urges a reconfiguration of how schools conceptualize participation, support, and shared responsibility. While the review identifies promising developments toward more relational and contextually attuned practices, it is constrained by the geographic and methodological scope of the current evidence. Further research is needed in underrepresented settings, grounded in the voices of young people, if the potential of school-based care is to be realized.

## Figures and Tables

**Table 1:** Categorization of included studies by chronic condition and intervention type.

#	Study (Author, Year)	Country	Condition	Design	Intervention	Outcomes
1	Kocaaslan et al. [15]	Türkiye	Asthma	** RCT	Animated asthma education video	* ↑ Inhaler technique, ↑ asthma knowledge
2	Halterman et al. [16]	USA	Asthma	RCT	SB-TEAM: Telemedicine + DOT	↓ Symptom days, ↓ urgent visits
3	Al-Sheyab et al. [17]	Jordan	Asthma	RCT	Peer-led asthma education	↑ Smoking resistance, ↑ asthma awareness
4	Kawafha & Tawalbeh [18]	Jordan	Asthma	Quasi-experimental	Teacher asthma education	↑ Knowledge, ↑ attitudes toward asthma care
5	Clark et al. [19]	USA	Asthma	RCT	Self-management program	↑ Asthma control, ↑ self-efficacy
6	McGovern et al. [20]	USA	Asthma and Anxiety	RCT	CBT and breathing retraining	↓ Anxiety, ↑ quality of life
7	Trivedi et al. [21]	USA	Asthma	Retrospective cohort/Intervention evaluation	School nurse-supervised daily inhaled corticosteroid program	↓ ED visits, ↓ hospital admissions, ↓ rescue med use, ↑ medication adherence
8	Cicutto et al. [22]	Canada	Asthma	Quasi-experimental	Nurse-led education	↑ Inhaler use, ↑ attendance
9	Szefler et al. [23]	USA	Asthma	Hybrid type 2 effectiveness-implementation trial	Building Bridges for Asthma Care (BBAC) school-based asthma program	↑ Asthma control, ↑ care plan completion, ↓ absenteeism, ↓ emergency visits
10	Smith et al. [24]	USA	Diabetes	Quasi-experimental	Staff workshop training	↑ knowledge ↑ confidence in emergency response
11	Al-Daghri et al. [25]	Saudi Arabia	Diabetes	RCT	Online diabetes course	↑ Teacher confidence, ↑ diabetes care skills
12	Peery et al. [26]	USA	Diabetes	Quasi-experimental	Nurse-led management	↑ Student self-efficacy, ↑ parent-teacher trust
13	Alreshidi et al. [27]	Saudi Arabia	Asthma	Quasi-experimental, pretest-posttest	School-based, nurse-delivered asthma education program	↑ Knowledge, ↑ quality of life, ↓ absenteeism, no change in attitude or long-term anxiety
14	Eakin et al. [28]	USA	Asthma	RCT	Head Start-based program	↑ Attendance, ↑ asthma control
15	Harris et al. [13]	UK	Asthma	RCT	Theatre-based asthma education and self-management workshop	↑ Knowledge, ↑ perception, feasibility, acceptability
16	Alkhotani et al. [29]	Saudi Arabia	Epilepsy	Quasi-experimental	Health education on seizure first aid for teachers	↑ Knowledge of seizure first aid
17	Eze et al. [30]	Nigeria	Epilepsy	RCT	Teacher epilepsy training	↓ Bias, ↑ preparedness
18	Sibley et al. [31]	Mexico	ADHD	RCT	Multi-tier behavior therapy	↓ ADHD symptoms, ↑ organization
19	Evans et al. [32]	USA	ADHD	RCT	After-school skills training vs. mentoring	↑ Behavior, ↑ academic outcomes (skills group)
20	DuPaul et al. [33]	USA	ADHD	Quasi-experimental	Teacher consultation + home-school planning	↑ Teacher–parent collaboration, ↓ inattention
21	Pfiffner et al. [34]	USA	ADHD	RCT	Collaborative Life Skills	↓ Oppositional behavior, ↑ academic engagement
22	Sibley et al. [35]	USA	ADHD	RCT	Family-centered CBT	↑ Time management, ↓ functional impairment
23	Langberg et al. [36]	USA	ADHD	RCT	HOPS executive function training	↑ Homework completion, ↑ planning
24	Kasari et al. [37]	USA	Autism	RCT	Peer social skills program	↓ Isolation, ↑ peer interaction
25	Locke et al. [38]	USA	Autism	Quasi-experimental	Playground peer facilitation	↑ Inclusion, ↑ social interaction
26	Thompson et al. [39]	USA	Cancer (re-entry)	RCT	Re-entry support	↑ Emotional wellbeing, ↑ reintegration outcomes
27	Kourosh et al. [40]	USA	Food Allergy	Quasi-experimental	Staff training	↑ Emergency response, ↑ staff confidence
28	Douma et al. [41]	Netherlands	Chronic illness	RCT	Op Koers Online (online psychosocial group intervention)	↑ Disease-related coping, ↑ HRQoL (social, school, psychosocial functioning)

Note: * ↑ = improvement/increase; ↓ = reduction/decrease. ** RCT = randomized controlled trial; CBT = cognitive behavioral therapy; DOT = directly observed therapy. The study selection and screening process is illustrated in the PRISMA 2020 flow diagram (Figure 1), and a summary of methodological quality is presented in Section 3.7 and detailed in Supplementary S2 (Quality appraisal summary).

**Table 6:** Summary of risk of bias (RoB 2) and JBI checklist quality appraisal for included studies.

Design	Tool Used	n	Low Risk	Some Concerns	High Risk
Randomized Controlled Trial	RoB 2 (Cochrane)	18	7	11	0
Quasi-experimental	JBI Checklist (JBI)	10	0	3	7

Notes: Most RCTs were robust in randomization and outcome measurement but faced common challenges, such as a lack of blinding and limited follow-up. Quasi-experimental designs, while offering valuable contextual insights, were often limited by methodological constraints in the absence of randomization and the use of single-group designs. No studies were excluded based on quality; instead, methodological limitations were considered when interpreting study findings. See Supplementary S2 for a full breakdown and study-level notes.

## Data Availability

Not applicable.

## Supplementary Materials

The following supporting information can be downloaded at: https://www.mdpi.com/article/10.3390/healthcare13161968/s1, S1: Database-specific search strategies. S2: Quality appraisal of included studies using RoB 2 and JBI tools.

## References

[1] Daly B.P., Litke S., Kiely J., Jones P.C., Wargel K., Flaspohler P., Mancini K. (2022). Effectively supporting youth with chronic illness in schools: External partnerships and training recommendations. Pediatr. Clin. N. Am..

[2] Sentenac M., Santos T., Augustine L., Michelsen S.I., Movsesyan Y., Ng K., Małkowska-Szkutnik A., Godeau E. (2023). Chronic health conditions and school experience in school-aged children in 19 European countries. Eur. Child Adolesc. Psychiatry.

[3] Runions K.C., Vithiatharan R., Hancock K., Lin A., Brennan-Jones C.G., Gray C., Payne D. (2020). Chronic health conditions, mental health and the school: A narrative review. Health Educ. J..

[4] Wahlström J., Låftman S.B., Modin B., Löfstedt P. (2021). Psychosocial Working Conditions in School and Life Satisfaction among Adolescents in Sweden: A Cross-Sectional Study. Int. J. Environ. Res. Public Health.

[5] Mejía-Rodríguez A.M., Kyriakides L. (2022). What matters for student learning outcomes? A systematic review of studies exploring system-level factors of educational effectiveness. Rev. Educ..

[6] Yu T., Xu J., Jiang Y., Hua H., Zhou Y., Guo X. (2022). School educational models and child mental health among K-12 students: A scoping review. Child Adolesc. Psychiatry Ment. Health.

[7] Lin G., Werner K., Alqunaiebet A., Hamza M.M., Alkanhal N., Alsukait R.F., Alruwaily A., Rakic S., Cetinkaya V., Herbst C.H. (2024). The cost-effectiveness of school-based interventions for chronic diseases: A systematic review. Cost Eff. Resour. Alloc. C/E.

[8] Foucault M. (1977). Discipline and Punish: The Birth of the Prison.

[9] Freire P. (1970). Pedagogy of the Oppressed.

[10] Slee R. (2011). The Irregular School: Exclusion, Schooling, and Inclusive Education.

[11] Noddings N. (2005). The Challenge to Care in Schools: An Alternative Approach to Education.

[12] Lundy L., McEvoy L. (2012). Children’s rights and research processes: Assisting children to (in)formed views. Childhood.

[13] Harris K., Newby C., Mosler G., Steed L., Griffiths C., Grigg J. (2022). School-based self-management intervention using theatre to improve asthma control in adolescents: A pilot cluster-randomised controlled trial. Pil Feasib Stud..

[14] Hoffmann T.C., Glasziou P.P., Boutron I., Milne R., Perera R., Moher D., Barbour V., Johnston M., Lamb S.E., Dixon-Woods M. (2014). Better reporting of interventions: Template for intervention description and replication (TIDieR) checklist and guide. BMJ.

[15] Kocaaslan E.N., Kostak M.A., Özdemir P.G. (2025). Effect of animated video education designed for children with asthma on asthma management and quality of life: A randomized controlled trial. Child Health Care.

[16] Halterman J.S., Fagnano M., Tajon R.S., Tremblay P., Wang H., Butz A., Perry T.T., McConnochie K.M. (2018). Effect of the School-Based Telemedicine Enhanced Asthma Management (SB-TEAM) Program on Asthma Morbidity: A Randomized Clinical Trial. JAMA Pediatr..

[17] Al-sheyab N., Gallagher R., Crisp J., Shah S. (2012). Peer-led education for adolescents with asthma in Jordan: A cluster-randomized controlled trial. Pediatrics.

[18] Kawafha M.M., Tawalbeh L.I. (2014). The effect of asthma education program on knowledge of school teachers: A randomized controlled trial. West. J. Nurs. Res..

[19] Clark N.M., Shah S., Dodge J.A., Thomas L.J., Andridge R.R., Little R.J. (2010). An evaluation of asthma interventions for preteen students. J. Sch. Health.

[20] McGovern C.M., Arcoleo K., Melnyk B. (2019). COPE for asthma: Outcomes of a cognitive behavioral intervention for children with asthma and anxiety. Sch. Psychol..

[21] Trivedi M., Patel J., Lessard D., Kremer T., Byatt N., Phipatanakul W., Pbert L., Goldberg R. (2018). School nurse asthma program reduces healthcare utilization in children with persistent asthma. J. Asthma.

[22] Cicutto L., To T., Murphy S. (2013). A randomized controlled trial of a public health nurse-delivered asthma program to elementary schools. J. Sch. Health.

[23] Szefler S.J., Cicutto L., Brewer S.E., Gleason M., McFarlane A., DeCamp L.R., Brinton J.T., Huebschmann A.G. (2022). Applying dissemination and implementation research methods to translate a school-based asthma program. J. Allergy Clin. Immunol..

[24] Smith C.T., Chen A.M., Plake K.S., Nash C.L. (2012). Evaluation of the impact of a diabetes education curriculum for school personnel on disease knowledge and confidence in caring for students. J. Sch. Health.

[25] Al-Daghri N.M., Amer O.E., Hameidi A., Alfawaz H., Alharbi M., Khattak M.N.K., Alnaami A.M., Aljohani N.J., Alkhaldi G., Wani K. (2022). Effects of a 12-Month Hybrid (In-Person + Virtual) Education Program in the Glycemic Status of Arab Youth. Nutrients.

[26] Peery A.I., Engelke M.K., Swanson M.S. (2012). Parent and teacher perceptions of the impact of school nurse interventions on children’s self-management of diabetes. J. Sch. Nurs..

[27] Alreshidi N.M., Livesley J., Al-Kalaldeh M., Long T. (2020). The impact of a school-based, nurse-delivered asthma health education program on quality of life, knowledge, and attitudes of Saudi children with asthma. Compr. Child Adolesc. Nurs..

[28] Eakin M.N., Zaeh S., Eckmann T., Ruvalcaba E., Rand C.S., Hilliard M.E., Riekert K.A. (2020). Effectiveness of a home- and school-based asthma educational program for Head Start children with asthma: A randomized clinical trial. JAMA Pediatr..

[29] Alkhotani A.M., Alkhotani A.M. (2022). Effect of health education on female primary school teachers’ knowledge of seizure first aid: An interventional study. Epilepsy Behav..

[30] Eze C.N., Ebuehi O.M., Brigo F., Otte W.M., Igwe S.C. (2015). Effect of Health Education on Trainee Teachers’ Knowledge, Attitudes, and First Aid Management of Epilepsy: An Interventional Study. Seizure.

[31] Sibley M.H., Graziano P.A., Coxe S.J., Bickman L., Martin P., Flores S. (2023). A randomized community-based trial of behavior therapy vs. usual care for adolescent ADHD: Secondary outcomes and effects on comorbidity. Beh Ther..

[32] Evans S.W., Owens J.S., Bunford N. (2014). Evidence-Based Psychosocial Treatments for Children and Adolescents with Attention-Deficit/Hyperactivity Disorder. J. Clin. Child Adolesc. Psychol..

[33] DuPaul G.J., Chronis-Tuscano A., Danielson M.L., Visser S.N. (2019). Predictors of receipt of school services in a national sample of youth with ADHD. J. Atten. Disord..

[34] Pfiffner L.J., Villodas M., Kaiser N., Rooney M., McBurnett K. (2013). Educational outcomes of a collaborative school–home behavioral intervention for ADHD. Sch. Psychol. Q..

[35] Sibley M.H., Graziano P.A., Kuriyan A.B., Coxe S., Pelham W.E., Rodriguez L., Sanchez F., Derefinko K., Helseth S., Ward A. (2016). Parent-teen behavior therapy + motivational interviewing for adolescents with ADHD. J. Consul Clin. Psychol..

[36] Langberg J.M., Epstein J.N., Becker S.P., Girio-Herrera E., Vaughn A.J. (2012). Evaluation of the Homework, Organization, and Planning Skills (HOPS) Intervention for Middle School Students with Attention Deficit Hyperactivity Disorder as Implemented by School Mental Health Providers. Sch. Psychol. Rev..

[37] Kasari C., Rotheram-Fuller E., Locke J., Gulsrud A. (2011). Making the Connection: RCT of a Peer-Mediated Intervention for Elementary School Children with Autism. J. Child Psychol. Psychiatry.

[38] Locke J., Kang-Yi C., Pellecchia M., Mandell D.S. (2019). It’s messy but real: A pilot study of the implementation of a social engagement intervention for children with autism in schools. J. Res. Spec. Educ. Needs.

[39] Thompson A.L., Christiansen H.L., Elam M., Hoag J.A., Irwin M.K., Pao M., Vollmer L.L., Patenaude A.F. (2015). Academic Continuity and School Reentry Support as a Standard of Care in Pediatric Oncology. Pediatr. Blood Cancer.

[40] Kourosh A., Nsobundu C.K., Khosla R., Guffey D., Minard C.G., Levinson A.J., Davis C.M. (2020). The effects of school staff food allergy education in a large urban school district. Health Behav. Policy Rev..

[41] Douma M., Maurice-Stam H., Gorter B., Houtzager B.A., Vreugdenhil H.J.I., Waaldijk M., Wiltink L., Grootenhuis M.A., Scholten L. (2021). Online psychosocial group intervention for adolescents with a chronic illness: A randomized controlled trial. Internet Interv..

[42] McGhan S.L., Wong E., Sharpe H.M., Hessel P.A., Mandhane P., Boechler V.L., Befus A.D. (2010). A children’s asthma education program: Roaring Adventures of Puff (RAP), improves quality of life. Can. Respir. J..

[43] Chadi N., Weisbaum E., Malboeuf-Hurtubise C., Kohut S.A., Viner C., Palaniyar N., Vo D.X. (2019). In-person vs. eHealth mindfulness-based intervention for adolescents with chronic illnesses: A pilot randomized trial. Adolesc. Psychiatry.

[44] Liptzin D.R., Gleason M.C., Cicutto L.C., Cleveland C.L., Shocks D.J., White M.K., Faino A.V., Szefler S.J. (2016). Developing, implementing, and evaluating a school-centered asthma program: Step-Up Asthma Program. J. Allergy Clin. Immunol. Pract..

