# Supplementary material for: A Systematic Review of School-Based Behavioral Interventions and the Symbolic Labor of Inclusion for Children with Chronic Illness
Source: Healthcare (Basel). 2025 Aug 11;13(16):1968. doi: 10.3390/healthcare13161968 (PMC12385309; doi:10.3390/healthcare13161968)
Supplement: Supplementary file 1 [file healthcare-13-01968-s001.zip › healthcare-3703026_ S2_Proofreading Updated.pdf]

Quality appraisal of included studies using RoB 2 and JBI tools.

Study (Author, Year)	Study design	Risk tool used	Overall quality rating	Notes
**Kocaaslan et al. (2025)[15]**	Randomized Controlled Trial (RCT)	RoB 2 (Cochrane Risk of Bias 2)	Some concerns	Unblinded educational intervention for asthma; potential performance bias due to lack of blinding, but otherwise a well-conducted trial.
**Halterman et al. (2018)[16]**	Cluster-RCT (School-based trial)	RoB 2	Low risk	Large cluster-RCT of a telemedicine asthma program; rigorous design with objective outcome measures (e.g., symptom-free days, ED visits), minimizing bias.
**Al-Sheyab et al. (2012)[17]**	Cluster-RCT (Educational intervention)	RoB 2	Some concerns	Cluster-randomized trial of a peer-led asthma education program; some risk of bias from lack of blinding and cluster allocation, but methods and follow-up were generally robust.
**Kawafha & Tawalbeh (2014)[18]**	Cross-sectional survey (pre-intervention)	JBI Checklist (Observational)	High risk	Descriptive study of Jordanian teachers' asthma management knowledge; no intervention or control group, so high risk of bias due to self-report and potential selection bias.
**Clark et al. (2010)[19]**	Randomized Controlled Trial (School-based)	RoB 2	Low risk	Self-management program for preteen students with asthma; demonstrated improved asthma control and self-efficacy. Well-designed with randomization and objective outcome measures, minimal bias risk.
**McGovern et al. (2022)[20]**	Quasi-experimental (Pilot study)	JBI Checklist (Quasi-experimental)	High risk	Uncontrolled pilot study (CBT+education for asthma-anxiety); no control group and small sample, so results are prone to confounding and observer bias.
**Trivedi et al. (2018)[21]**	Quasi-experimental (Implementation pilot)	JBI Checklist	High risk	Implementation pilot of school-supervised asthma therapy; no randomized control, outcomes observational, leading to high risk of bias (feasibility focus with uncontrolled design).
**Cicutto et al. (2013)[22]**	Cluster-RCT (School nurse-led program)	RoB 2	Low risk	Large RCT of a nurse-delivered asthma education program; well-designed (130 schools) with strong methodology. Minor performance

Quality appraisal of included studies using RoB 2 and JBI tools.

				bias (unblinded schools) but overall low risk of bias.
**Szefler et al. (2019)[23]**	RCT (Multisite trial)	RoB 2	Low risk	Well-conducted multisite RCT of a coordinated asthma care intervention; robust randomization and outcome assessment (e.g., medication adherence, lung function), yielding low risk of bias.
**Smith et al. (2012)[24]**	Quasi-experimental (Pre–post study)	JBI Checklist	High risk	One-group pre/post evaluation of a diabetes education workshop for school staff; no control group and reliance on self-reported confidence gains, resulting in high risk of bias.
**Al-Daghri et al. (2022)[25]**	Quasi-experimental (Pre–post study)	JBI Checklist	High risk	Uncontrolled 12-month school-based intervention for type1 diabetes; no randomization or comparison group, so improvements in glycemic control could be due to confounders (high risk of bias).
**Peery et al. (2012)[26]**	Observational (Descriptive study)	JBI Checklist	High risk	Descriptive study of school nurse interventions in diabetes (no interventional control); high risk of bias due to absence of a control group and use of subjective parent/teacher perceptions.
**Alreshidi et al. (2020)[27]**	Quasi-experimental (Pretest–posttest)	JBI Checklist	Some concerns	School-based, nurse-delivered asthma health education program; improved asthma knowledge and quality of life, reduced absenteeism, but no change in attitudes or long-term anxiety. Some bias concerns due to non-randomized design and self-reported outcomes.
**Eakin et al. (2020)[28]**	RCT (Parallel-group trial)	RoB 2	Low risk	Randomized trial of home- and school-based asthma education (Head Start program); strong design with adequate sample size and objective outcomes (improved asthma control), low overall bias risk.
**Harris et al. (2022)[13]**	Pilot cluster-randomised controlled trial (School-based)	RoB 2	Some concerns	Theatre-based asthma education and self-management workshop for adolescents; increased asthma knowledge and perception of self-management. Pilot nature limits

Quality appraisal of included studies using RoB 2 and JBI tools.

				statistical power; some bias risk due to lack of blinding.
**Alkhotani et al. (2022)[29]**	Quasi-experimental (Pre–post interventional study)	JBI Checklist	Some concerns	Health education on seizure first aid for female primary school teachers; significantly improved knowledge of seizure first aid. Some risk of bias due to non-randomized design, lack of blinding, and reliance on self-reported outcomes.
**Eze et al. (2015)[30]**	Quasi-experimental (Pre–post study)	JBI Checklist	High risk	Single-group educational intervention for trainee teachers on epilepsy; high risk of bias with no control group and outcomes based on before-and-after knowledge assessments.
**Sibley et al. (2023)[31]**	RCT (Parallel-group trial)	RoB 2	Some concerns	RCT comparing a multi-component behavioral therapy to usual care for adolescent ADHD; some concerns due to lack of blinding (participants and personnel aware of treatment) and reliance on parent/teacher-reported outcomes.
**Evans et al. (2014)[32]**	RCT (Parallel-group trial)	RoB 2	Some concerns	RCT of an after-school skills coaching program for adolescents with ADHD; moderate risk of bias due to lack of blinding and notable attrition (attendance challenges), but the randomized design supports a fair comparison.
**DuPaul et al. (2011)[33]**	RCT (Parallel-group trial)	RoB 2	Some concerns	RCT of a teacher consultation and daily report card intervention for ADHD; some risk of bias (unblinded teachers and limited sample) though the study used random assignment and standard outcome measures in school settings.
**Pfiffner et al. (2016)[34]**	RCT (Parallel-group trial)	RoB 2	Some concerns	RCT of the Collaborative Life Skills (CLS) training for ADHD; moderate sample. No blinding of participants or outcome reporters, raising some bias concerns, but otherwise a well-implemented trial.
**Sibley et al. (2016)[35]**	RCT (Parallel-group trial)	RoB 2	Some concerns	RCT of a family-centered behavioral intervention for adolescents with ADHD; some concerns due to lack of blinding

Quality appraisal of included studies using RoB 2 and JBI tools.

				and relatively small sample size, but randomization and outcome measurement were acceptable.
**Langberg et al. (2018)[36]**	RCT (Parallel-group trial)	RoB 2	Some concerns	RCT of the HOPS organizational skills intervention for middle-school students with ADHD; some risk of bias due to unblinded delivery and mainly subjective outcomes (teacher reports of homework completion), though randomized design limits systematic bias.
**Kasari et al. (2012)[37]**	RCT (Parallel-group trial)	RoB 2	Some concerns	RCT of a peer-mediated social inclusion intervention for children with autism; generally rigorous, with blinded assessors for some social outcome measures, but some concerns remain (e.g., possible knowledge of group assignment among staff/peers).
**Locke et al. (2013)[38]**	Quasi-experimental (Pilot intervention)	JBI Checklist	High risk	Pilot study of the "Remaking Recess" playground inclusion program for autism; no randomized control (pre- and post-intervention comparisons only), so high risk of bias due to potential confounding and lack of blinding.
**Thompson et al. (2015)[39]**	Quasi-experimental (Program evaluation)	JBI Checklist	High risk	Program implementation report on school reentry support for pediatric cancer survivors; no control group or experimental design (observational data only), yielding high risk of bias.
**Kourosh et al. (2020)[40]**	Quasi-experimental (Multigroup evaluation)	JBI Checklist	Some concerns	Quasi-experimental evaluation of a food allergy education program (comparing online vs in-person training modes); some bias concerns due to non-random group assignment and reliance on staff knowledge gains, although pre/post improvements were measured for each group.
**Douma et al. (2021)[41]**	RCT (Parallel-group trial)	RoB 2	Low risk	Online psychosocial group intervention ("Op Koers Online") for adolescents with chronic illness; demonstrated significant improvements in disease-related coping and HRQoL (social, school, psychosocial functioning). Strong randomization and blinded

Quality appraisal of included studies using RoB 2 and JBI tools.

				assessment for some outcomes; minimal risk of bias.
