# Supplementary material for: A Systematic Review of School-Based Behavioral Interventions and the Symbolic Labor of Inclusion for Children with Chronic Illness
Source: Healthcare (Basel). 2025 Aug 11;13(16):1968. doi: 10.3390/healthcare13161968 (PMC12385309; doi:10.3390/healthcare13161968)
Supplement: Supplementary file 1 [file healthcare-13-01968-s001.zip › healthcare-3703026_S1_Database-specific search strategies.pdf]

Database-specific search strategies

(Search period: January 2010 - April 2025)

PubMed (searched April 2025)

("chronic disease"[Title/Abstract] OR asthma[MeSH Terms] OR diabetes[MeSH Terms] OR epilepsy[MeSH Terms] OR ADHD[MeSH Terms]) AND

("school-based"[Title/Abstract] OR "inclusive education"[Title/Abstract] OR "special education"[Title/Abstract]) AND

("intervention"[Title/Abstract] OR "self-management"[Title/Abstract] OR "psychosocial"[Title/Abstract]) AND

("children"[Title/Abstract] OR "adolescents"[Title/Abstract])

ERIC (searched April 2025)

("chronic illness" OR asthma OR diabetes OR epilepsy OR ADHD) AND

("school-based" OR "inclusive education" OR "special education") AND

("intervention" OR "self-management" OR "psychosocial") AND

("children" OR "adolescents")

PsycINFO (searched April 2025)

("chronic illness" OR asthma OR diabetes OR epilepsy OR ADHD).ti,ab. AND

("school-based" OR "inclusive education" OR "special education").ti,ab. AND

("intervention" OR "self-management" OR "psychosocial").ti,ab. AND

("children" OR "adolescents").ti,ab.

Scopus (searched April 2025)

TITLE-ABS-KEY("chronic illness" OR asthma OR diabetes OR epilepsy OR ADHD) AND

TITLE-ABS-KEY("school-based" OR "inclusive education" OR "special education") AND

TITLE-ABS-KEY("intervention" OR "self-management" OR "psychosocial") AND

TITLE-ABS-KEY("children" OR "adolescents")

Web of Science (searched April 2025)

TS=("chronic illness" OR asthma OR diabetes OR epilepsy OR ADHD) AND

TS=("school-based" OR "inclusive education" OR "special education") AND

TS=("intervention" OR "self-management" OR "psychosocial") AND

TS=("children" OR "adolescents")
